# Mutational Profile and Retinal Phenotypes of *PCARE*-Related Cone-Rod Dystrophies in a Mexican Cohort

**DOI:** 10.1155/2024/4003914

**Published:** 2024-03-04

**Authors:** Víctor R. López-Rodríguez, Rocío Arce-González, Alan Martínez-Aguilar, Carlos E. Rodríguez-López, Sergio Groman-Lupa, M. Isabel Neria-González, Genaro Rodríguez-Uribe, Juan C. Zenteno

**Affiliations:** ^1^Department of Genetics, Institute of Ophthalmology “Conde de Valenciana”, Mexico City, Mexico; ^2^Retinal Dystrophies Clinic, Institute of Ophthalmology “Conde de Valenciana”, Mexico City, Mexico; ^3^Retina Department, Institute of Ophthalmology “Conde de Valenciana”, Mexico City, Mexico; ^4^CODET Vision Institute, Tijuana, Mexico; ^5^Laboratory of Integrative Microbiology and Molecular Biology, Division of Chemical and Biochemical Engineering, TecNm: Tecnológico de Estudios Superiores de Ecatepec, Ecatepec de Morelos, Estado de México, Mexico; ^6^Facultad de Medicina y Psicología, Universidad Autónoma de Baja California, Tijuana, Mexico; ^7^Department of Biochemistry, Faculty of Medicine, UNAM, Mexico City, Mexico; ^8^Rare Disease Diagnostic Unit, Faculty of Medicine, National Autonomous University of Mexico (UNAM), Mexico City, Mexico

## Abstract

**Purpose:**

The aim of the study is to describe the genotype and phenotype of a Mexican cohort with *PCARE*-related retinal disease.

**Methods:**

The study included 14 patients from 11 unrelated pedigrees with retinal dystrophies who were demonstrated to carry biallelic pathogenic variants in *PCARE*. Visual assessment methods included best corrected visual acuity, color fundus photography, Goldmann visual field test, kinetic perimetry, dark/light adapted chromatic perimetry, full-field electroretinography, autofluorescence imaging, and spectral domain-optical coherence tomography imaging. Genetic screening was performed either by gene panel sequencing or by exome sequencing.

**Results:**

According to the results of multimodal imaging and functional tests, all 14 patients were diagnosed with cone-rod dystrophy. Six different *PCARE* pathogenic alleles were identified in our cohort, including three novel mutations: c.3048_3049del (p.Tyr1016^*∗*^), c.3314_3315del (p.Ser1105^*∗*^), and c.551A > G (p.His184Arg). Notably, alleles p.His184Arg, p.Arg613^*∗*^, and p.Arg984^*∗*^ were present in 18 of the 22 (82%) *PCARE* alleles from probands in our cohort.

**Conclusion:**

Our work expands the *PCARE* mutational profile by identifying three novel pathogenic variants causing retinal dystrophy. While phenotypic variations occurred among patients, a cone-rod dystrophy pattern was observed in all affected individuals.

## 1. Introduction

Inherited retinal degenerations (IRDs) are a heterogeneous group of genetic disorders characterized by progressive dysfunction of neuroretinal cells and/or the retinal pigment epithelium. IRDs have a combined prevalence of approximately one case per 2,000 individuals [1] and are considered the most common cause of blindness in the working-age population [2]. IRDs are a paradigmatic example of diseases with phenotypic and genotypic variabilities, as their age of onset, severity of clinical manifestations, and rate of visual loss are considerably variable among individuals and as mutations in over 280 genes can result in the disease [3, 4]. IRD-related genes participate in a myriad of cellular processes that are essential for the structural and functional integrity of retinal tissue [5]. Genetic screening of individuals with IRDs is a crucial procedure for accurate diagnosis, prognosis, and management of patients, and it has been tremendously improved in recent years by the widespread incorporation of next-generation sequencing (NGS) techniques [6, 7].

Pathogenic variants in *PCARE*, a gene located at 2p23.2 and previously known as C2orf71, has been recently demonstrated in IRDs [8, 9]. Recessive loss-of-function variants in this gene have been reported in subjects with different retinal phenotypes involving both rods and cones, namely retinitis pigmentosa (RP) and cone-rod dystrophy (CRD) [10–13]. *PCARE*-related IRD is associated with wide variation in terms of age of onset and progression rate of retinal damage with vision loss typically beginning in the second or third decade of life; about half of the patients also present with nyctalopia at the time of consultation, and vision can be preserved until the fifth or sixth decade [11]. Fundus appearance is particularly variable, ranging from early-onset maculopathy to changes resembling retinitis pigmentosa [11]. The prevalence of *PCARE*-related IRD is currently unknown, but it is expected to vary from 1% as observed in a French cohort [14] to 15% in a Swiss population [10].

To date, more than 30 *PCARE* pathogenic variants have been recognized in patients suffering from diverse retinal phenotypes, most of them from European and Asian ethnicities [15, 16]. The majority of these variants correspond to single nucleotide variants or indels that generate premature stop codons or frameshifts [17].

The aim of this study was to describe the phenotypes and disease-causing variants in a cohort of *PCARE*-related IRD patients from Mexico. Our results add to the knowledge of the clinical and molecular spectrum of retinal dysfunction due to *PCARE* mutations.

## 2. Materials and Methods

### 2.1. Study Population

The study corresponded to a retrospective and descriptive case series comprising 14 affected patients with retinal disease who were demonstrated to carry causative variants in *PCARE*; all included patients were of Mexican origin. The study was approved by the Institutional Review Board of the Institute of Ophthalmology “Conde de Valenciana” in Mexico City. All procedures adhered to the tenets of the Declaration of Helsinki, and written informed consent was obtained from the participants.

### 2.2. Clinical Data

Medical records were reviewed to collect clinical data and participants underwent a complete eye examination, including visual acuity testing, ultrawide fundus photography, Goldmann visual field kinetic perimetry, dark/light adapted chromatic perimetry (Metrovision, MonCvONE, France), autofluorescence imaging (FAF), spectral domain-optical coherence tomography (SD-OCT) (Spectralis; Heidelberg Engineering, Heidelberg, Germany), and full-field electroretinography (ffERG) (Metrovision, MonPackONE, France). Mean deficits in chromatic perimetry scores were calculated using an internal database of values from normal Mexican population. The ERG protocol complied with the standards of the International Society for Clinical Electrophysiology of Vision.

### 2.3. Genetic Analysis

*PCARE*disease-causing mutations were identified by either gene panel (patients #1–9, 14) or exome sequencing (ES) (patients #10–13). Briefly, genomic DNA (gDNA) was extracted from peripheral blood leukocytes using the QIAamp DNA Blood Mini Kit (Qiagen, Germany), following the manufacturer's protocol. In patients #1–9, and 14 gDNA was enriched for targeted regions using a hybridization-based protocol and sequenced using Illumina technology. Sequence analysis and deletion/duplication testing were performed on 298 genes included in the Invitae Inherited Retinal Disorders Panel (Invitae, San Francisco, CA). Target regions were sequenced with ≥50x depth, and reads were aligned to the GRCh37/Hg19 reference sequence. In patients #10–13, ES was performed at 3Billion, Inc. (Seoul, South Korea). DNA library preparation was performed using the IDT xGen Exome Research Panel v2.0 kit (Integrated DNA Technologies, Coralville, Iowa, USA), and sequenced on NovaSeq 6000 (Illumina, San Diego, CA, USA). The mean depth-of-coverage was 140x with a minimum of 98.5% of the targeted region covered at 30x. The base call (BCL) sequence files generated by NovaSeq 6000 were converted and demultiplexed to FASTQ files using bcl2fastq v2.20.0.422. Sequence reads in the FASTQ files were aligned to the human reference genome (GRCh37/hg19) using BWA-mem 0.7.17 to generate BAM files. BAM files were processed following the GATK best practices (GATK v.3.8) for variant calling to generate VCF files. Exome sequencing data annotation and variant filtration were performed using the Franklin platform (Genoox, Palo Alto, CA). The designation of pathogenic or likely pathogenic variants was performed according to the American College of Genetics and Genomics (ACMG) guidelines. In silico analysis and visualization of residue conservation and position at the protein level were performed using Jalview (jalview.org) and ConSurf-DB (consurfdb.tau.ac.il), respectively, in relevant missense variants. Variants of clinical significance were confirmed and segregated in families by Sanger sequencing. Primers sequences and PCR conditions are available on request.

## 3. Results

### 3.1. Patients' Summary

A total of 14 patients pertaining to 11 unrelated families suffering from *PCARE*-related retinal dystrophies were included in the analysis. Tables 1 and 2 present their demographic and clinical data; two families (II and V) with more than one affected subject were ascertained. The cohort included eight males (57%) and six females. The mean age of symptoms onset was 17.2 years (±11.05, range 5–36), and the mean age upon clinical examination was 36 years (±11.80, range 16–54). Family VI reported a history of consanguinity, while family IX reported endogamy.

### 3.2. Ocular Phenotypes

**Figure 1:**
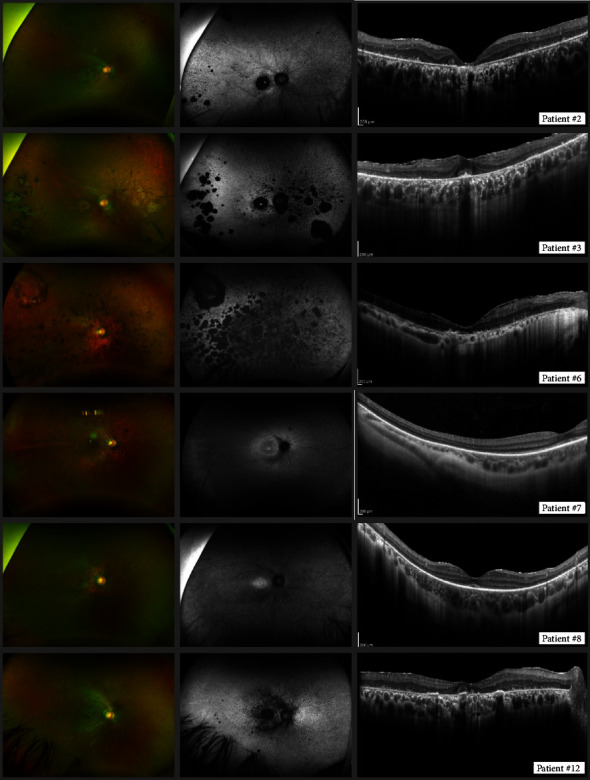
Retinal images of patients. The left column shows ultra-wide fundus photography, the middle column shows autofluorescence imaging, and the right column shows SD-OCT images. Every row corresponds to a different patient.

All patients had clinical data from at least one ophthalmologic examination, which revealed a tendency towards symmetric affectation (Tables 1 and 2). The symptoms at the onset of the disease were nyctalopia (8/14, 57%), vision loss (3/14, 21%), photophobia (2/14, 14%), and photopsia (1/14, 7%). Visual acuity at examination ranged from 0.09 logMAR to light perception, with 10 out of 13 available patients having in both eyes a logMAR value of 0.50 or worse. Funduscopic common findings included a tessellated appearance, hypopigmented dotting, optic disc pallor, and vessel attenuation (Figure 1). Atrophic maculopathy was observed in six patients (ID# 2, 3, 6, 11, 12, and 14). Chromatic perimetry (available in four patients, #7–9, 11) showed a reduction in the response of both rod and cone systems; photopic mean deficits ranged from 12.63 dB to 20.14 dB, and the scotopic mean deficits ranged from 20.08 dB to 41.9 dB. Five patients (ID# 2, 3, 6, 12, and 13) with low visual acuity or poor fixation were unable to perform a chromatic perimetry test, so a full-field stimulus threshold test (FST) was completed instead. The FST showed a reduced response under scotopic conditions and a more reduced response under photopic conditions in all these patients (Table 3). Goldmann perimetry results were available in seven patients. Preservation of a central island of vision was noted in four patients (ID# 2, 3, 8, 10); additionally, patient #8 had a nasal island of vision in the periphery. In the youngest patient (ID# 7), the visual field was normal using V4e stimulus, while a perifoveal relative scotoma with an augmented peripapillary scotoma was found in the OD using the I4e stimulus.

In 11 patients in whom FAF test was performed, the most frequent findings were hypo-AF with a nummular or mottling pattern in the periphery (ID# 2, 3, 6–14); hyper-AF foveal dot (ID# 2, 3, 7–13); hypo-AF in the central macular area (ID# 2, 3, 6, 9–14); peripapillary hypo-AF (ID# 2, 3, 6, 9, 12, 13); and hyper-AF macular ring surrounding the mentioned hypo-AF macular area (ID# 3, 7–10, 14) (Figure 1). All ten patients who underwent full-field ERG had an abolished response in scotopic, mesopic, and photopic conditions, with the exception of patient #7 who had a subnormal scotopic and mesopic response in addition to an abolished photopic response (see supplementary Table (available here)).

SD-OCT images, available in 11 patients, demonstrated loss of outer retinal layers to different degrees in all of them, preservation of the ellipsoid zone (EZ) in four (ID# 7, 8, 10, 13), and severely atrophic damage with loss of retinal architecture in five patients (ID# 2, 3, 6, 11, and 12). Outer retinal tubulations (ORT) appeared in three patients (ID# 2, 3, 12) (Figure 1). According to multimodal imaging and functional assessment, all 14 patients were diagnosed with CRD.

### 3.3. Genetic Findings

**Figure 2:**
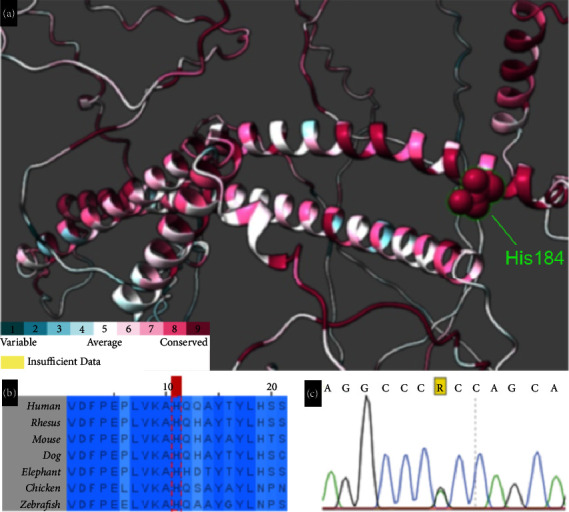
In silico analysis of the novel p.His184Arg variant in PCARE. (a) Close up of the predicted protein structure of PCARE, the p.His184 residue is located in a predicted helical coiled coil domain. (generated on ConSurf) (b) Protein sequence alignment of PCARE orthologs among different species; the red mark delimitates the p.His184 residue. (generated on Jalview) (c) Sanger sequencing chromatogram showing the c.551A > G (p.His184Arg) variant in the genomic DNA of one heterozygous patient.

All affected individuals carried biallelic *PCARE* pathogenic alleles, including a total of 6 distinct gene variants (Table 4). Pathogenic alleles included four nonsense (two novel: p.Tyr1016^*∗*^ and p.Ser1105^*∗*^), one frameshift, and one novel missense variant (p.His184Arg). Notably, three variants (p.His184Arg, p.Arg613^*∗*^, and p.Arg984^*∗*^) were present in 18 of the 22 *PCARE* alleles from the 11 probands (82%). Segregation analysis confirmed compound heterozygosity in four probands while a homozygous status was confirmed in the other seven. Based on the American College of Medical Genetics and Genomics criteria for variant assessment, all five identified null variants were classified as pathogenic, while the single identified novel missense variant (p.His184Arg) was classified as likely pathogenic. Of note, the p.His184Arg variant affects a highly conserved residue (GERP: 5.52) (Figure 2) and has an extremely low frequency (gnomAD 0.0004%), and *in silico* molecular analysis with different tools predicts a deleterious effect (SIFT: 0, PolyPhen-2: 1, VARITY: 0.92).

## 4. Discussion

In the present study, we describe in depth the clinical and genetic features of 14 patients, from 11 different pedigrees, with biallelic disease-causing *PCARE* variants. To our knowledge, this is one of the largest cohorts of *PCARE*-related IRDs reported to date and the first to include Latin American patients, a highly underrepresented population in published IRDs cohorts.

*PCARE* stands for photoreceptor cilium actin regulator, which is the name given to the protein product. As its name indicates, it is specifically expressed in the retina [8], and there is evidence showing its localization in the connecting cilium of photoreceptor cells where it participates, along with other proteins (e.g., WASF3), in actin dynamics for the expansion of the ciliary membrane, an important process for outer segment development and homeostasis [18–20].

In our cohort, the clinical findings showed a generalized photoreceptor disorder with early macular involvement, compatible with a CRD pattern. The clinical diagnosis of CRD was established mainly based on the results of chromatic perimetry, since there was a relatively better response under scotopic conditions in all the patients. Early macular atrophy and prominent cone-system deterioration have been mentioned in several reported cases of PCARE retinopathy, usually co-occurring with manifestations of rod-system deterioration [9–11, 14].

The descriptive data obtained from this cohort show, in general terms, a variable expression of the disease, including a variable age at onset ranging from the first to the fourth decade of life, and heterogeneity in the severity of visual symptoms. Indeed, nyctalopia as the first reported symptom in some patients is unexpected for a CRD diagnosis, and this could be the result of early and severe damage in regions with a high density of rods, including some regions of the macula, along with cone damage. All of our patients presented visual loss to some degree, progressing to light perception vision after the fourth decade of life; as expected, older individuals exhibited worse visual acuity in our cohort. Notably, clinical variation appeared to be less marked among subjects pertaining to the same family than among patients from unrelated pedigrees.

ORTs were observed on OCT images from three patients in our cohort. These are tubular retinal structures formed in the outer nuclear layer by reorganization of photoreceptors in different stages of degeneration, Müller cells and RPE cells [21]. These structures have been associated with a variety of retinal conditions, such as age-related macular degeneration, diabetic retinopathy, choroidopathies, and retinal dystrophies [22, 23]. Our observation of ORTs in PCARE-related IRD is consistent with previous reports [11, 15], and notably, the three subjects presented the ORTs in both eyes. In two cases, asymmetric ORTs in terms of their size were observed and, remarkably, in both cases, the eye with the larger ORTs presented better visual acuity (Table 1). Interestingly, a previously reported PCARE-related IRD case series described three patients with ORTs who appeared to have better visual acuity than the rest of the cohort [11]. Although the clinical significance of ORTs is controversial, some authors have recognized their potential use as clinical biomarkers of prognosis [24, 25].

The genetic characterization of our cohort identified a total of six different *PCARE* pathogenic variants, including three novel mutations. Although all the included families were apparently unrelated, certain *PCARE* variants had a noticeably higher frequency than others. Interestingly, two of these common variants, p.Arg613^*∗*^ and p.Arg984^*∗*^, have been reported in patients of Korean [11] and French [14] ethnicities, respectively, suggesting that these could be recurrent gene variations. In contrast, the p.His184Arg missense variant, observed in 4 out of 22 probands' alleles, had not been previously reported. While a possible explanation for this high frequency is a founder mutation effect in our population, additional haplotype analyses are required to confirm this hypothesis.

In accordance with previous reports, our results indicate that the majority of IRD-related *PCARE* variants are predicted null variants. Thus, five different null variants and a single missense variant were characterized in our cohort, with all six having a predicted loss-of-function effect. Of note, five out of six variants identified here introduce premature termination codons (PTCs), and according to the canonical rules known for nonsense-mediated mRNA decay activation [26], all of them are predicted to activate the mechanism leading to transcript degradation. On the other hand, mutation modeling shows that the novel p.His184Arg variant affects a highly conserved residue of the PCARE protein (Figure 2), located in a likely structured region corresponding to a helical coiled coil domain, which is predicted to have an important role in the function of the protein [18, 19]. Previously characterized pathogenic *PCARE* missense variants as p.Ile201Phe [8] and p.Cys599Arg [27] also affect relatively conserved residues. Even so, a clear genotype-phenotype correlation was not established in our cohort.

In conclusion, we report one of the largest cohorts of *PCARE*-related retinal dystrophy, and the first that includes Latino American population. Our results support that CRD is the main phenotype related to *PCARE* defects and confirm the association of ORT to the disease, an OCT finding that could serve as a clinical biomarker of the disorder. We also expand the mutational spectrum of *PCARE* with the report of three novel disease-causing variants.

## Figures and Tables

**Table 1:** Demographic, clinical features, and genetic variants of the *PCARE*-related Mexican cohort.

Subject #	Family	Sex	PCARE variants	Age at onset (years)	Age at exam (years)	First symptom	BCVA (LogMAR)		Clinical diagnosis	Chromatic perimetry/FST	ffERG		
							OD	OS			*P*	*M*	*S*
1	I	F	p.His184Arg/p.His184Arg	36	42	Visual loss	LP	LP	CRD	n/a	n/a		
2	II	F	p.His184Arg/p.Arg984^*∗*^	23	39	Nyctalopia	1.3	LP	CRD	FST *P*: *r* > *S*: *r*	*u*	*u*	*u*
3	II	M	p.His184Arg/p.Arg984^*∗*^	25	30	Nyctalopia	0.09	0.09	CRD	FST *P*: *r* > *S*: *r*	*u*	*u*	*u*
4	III	F	p.Arg984^*∗*^/p.Arg984^*∗*^	n/a	23	Visual loss	0.50	0.50	CRD	n/a	n/a		
5	IV	M	p.Asn316Metfs^*∗*^7/p.Asn316Metfs^*∗*^7	8	29	Vision loss	n/a		CRD	n/a	n/a		
6	V	M	p.Arg613^*∗*^/p.His184Arg	5	49	Nyctalopia	LP	LP	CRD	FST *P*: *r* > *S*: *r*	*u*	*u*	*u*
7	V	F	p.Arg613^*∗*^/p.His184Arg	6	16	Nyctalopia	0.17	0.17	CRD	CP *P*: *r* > *S*: *r*	*u*	*r*	*r*
8	V	M	p.Arg613^*∗*^/p.His184Arg	6	21	Nyctalopia	0.09	0.09	CRD	CP *P*: *r* > *S*: *r*	*u*	*u*	*u*
9	VI	F	p.Arg613^*∗*^/p.Arg613^*∗*^	17	23	Nyctalopia	1.17	1.30	CRD	CP *P*: *r* > *S*: *r*	*u*	*u*	*u*
10	VII	M	p.Arg984^*∗*^/p.Arg984^*∗*^	12	51	Photophobia	LP	LP	CRD	n/a	*u*	*u*	*u*
11	VIII	F	p.Arg613^*∗*^/p.Tyr1016^*∗*^	12	45	Photophobia	0.54	0.69	CRD	CP *P*: *r* > *S*: *r*	*u*	*u*	*u*
12	IX	M	p.Arg613^*∗*^/p.Arg613^*∗*^	8	39	Photopsias	0.60	LP	CRD	FST *P*: *r* > *S*: *r*	*u*	*u*	*u*
13	X	M	p.Arg984^*∗*^/p.Ser1105^*∗*^	30	54	Nyctalopia	1.47	LP	CRD	FST *P*: *r* > *S*: *r*	*u*	*u*	*u*
14	XI	M	p.Arg984^*∗*^/p.Arg984^*∗*^	36	43	Nyctalopia	LP	LP	CRD	n/a	n/a		

BCVA, best corrected visual acuity; CRD, cone-rod dystrophy; FST, full-field stimulus threshold; LP, light perception; *M*, mesopic; n/a, not available; OD, oculus dexter; OS, oculus sinister; *P*, photopic; *r*, reduced; *S*, scotopic; *u*, undetectable; >, more affected than.

**Table 2:** Ophthalmologic findings in the cohort.

Subject	Fundus appearance	FAF	SD-OCT	Goldmann perimetry
1	Peripheral bone spicules, retinal vessel attenuation, optic disc pallor, generalized hypopigmented dotting and peripheral pavingstone-like lesions	n/a	n/a	n/a

2	Peripheral isolated bone spicules, retinal vessel attenuation, optic disc pallor, generalized hypopigmented dotting and peripheral pavingstone-like lesions, circumscribed atrophy with diffuse pigmentation	Isolated hypo-AF with a nummular pattern in the periphery. Peripheral and macular hypo/hyper-AF mottling. Peripapillary hypo-AF. Central macular circumscribed hypo-AF, with a central hyper-AF dot	Epiretinal membrane. Widespread loss of outer retinal layers and RPE irregularities. Prominent foveal atrophy. Large perifoveal ORT (OD), perifoveal ORT (OS). Hyper-reflective deposits above the RPE	V4e: OU minimal central island of vision

3	Peripheral bone spicules, retinal vessel attenuation, optic disc pallor, generalized hypopigmented dotting and peripheral pavingstone-like lesions, circumscribed atrophy with diffuse pigmentation	Hypo-AF with a nummular pattern in the periphery. Peripheral and macular hypo/hyper-AF mottling. Peripapillary hypo-AF. Circumscribed central macular hypo-AF, surrounded by a hyper-AF ring, and a central hyper-AF dot	Epiretinal membrane. Widespread loss of outer retinal layers, thinning of inner retinal layers and RPE irregularities. Large foveal ORT (OU). Hyper-reflective deposits above the RPE	V4e: OU minimal central island of vision

4	Macular hypopigmented and hyperpigmented lesions	n/a	n/a	n/a

5	n/a	n/a	n/a	n/a

6	Peripheral bone spicules, retinal vessel attenuation, optic disc pallor, generalized hypopigmented dotting and peripheral pavingstone-like lesions, macular clumped pigmentation	Extensive hypo-AF with a nummular pattern in peripheral, peripapillary and macular areas. Hypo/hyper-AF mottling involving the periphery and macula. Circumscribed central macular hypo-AF	Epiretinal membrane. Widespread loss of outer retinal layers, thinning of inner retinal layers and RPE irregularities. Foveal atrophy. Hyper-reflective deposits above the RPE	n/a

7	Tessellated appearance, hypopigmented peripheral dotting	Peripheral hypo-AF mottling. Hyper-AF macular ring surrounding a normal-AF area with a central hyper-AF dot	Irregularities of outer retinal layers. Peripheral EZ disruption	V4e: OU normal I4e: OD increased peripheral relative scotoma, peripapillary scotoma; OS normal

8	Peripapillary atrophy, tessellated appearance, hypopigmented peripheral dotting	Peripheral hypo-AF mottling. Hyper-AF in the central macular area with a diffuse central hyper-AF dot	Widespread loss or outer retinal layers with parafoveal ELM preservation, and foveal EZ preservation	V4e: OD central island of vision, preservation of nasal-peripheral area; OS temporal constriction I4e: OD undetectable; OS central island of vision

9	Hypopigmented generalized dotting, macular clumped pigmentation	Peripheral and macular hypo/hyper-AF mottling. Peripapillary hypo-AF with adjacent diffuse nasal hyper-AF. Diffuse central macular hypo-AF, surrounded by a hyper-AF ring and with a central hyper-AF dot	Epiretinal membrane. Widespread loss of outer retinal layers and RPE irregularities. Hyper-reflective deposits above the RPE	V4e: OD superior constriction; OS concentric constriction I4e: OD minimal central island of vision; OS undetectable

10	Peripheral isolated bone spicules, retinal vessel attenuation, generalized hypopigmented dotting, macular circumscribed atrophy with diffuse pigmentation	Perivascular hypo-AF mottling. Hyper-AF macular ring surrounding a circumscribed central macular hypo-AF area. OD with a foveal hyper-AF dot	Widespread loss or outer retinal layers with foveal ELM and EZ preservation. Hyper-reflective deposits above the RPE	V4e: OD minimal central island of vision; OS central island of vision

11	Hypopigmented dotting in the retinal equator, vessel attenuation and macular clumped pigmentation	Peripheral hyper-AF mottling, and equatorial hypo-AF. Temporal peripapillary hyper-AF. Diffuse central macular hypo-AF, with a central hyper-AF dot	Epiretinal membrane. Widespread loss of outer retinal layers, thinning of inner retinal layers with some foveal preservation. Foveal atrophy. RPE irregularities. Hyper-reflective deposits above the RPE	V4e: OU annular scotoma

12	Peripheral bone spicules, retinal vessel attenuation, optic disc pallor, generalized hypopigmented dotting and peripheral pavingstone-like lesions	Isolated peripheral nummular hypo-AF. Peripheral and macular hypo/hyper-AF mottling. Peripapillary hypo-AF. Circumscribed central macular hypo-AF. OD with a central hyper-AF dot	Epiretinal membrane. Widespread loss of outer retinal layers, thinning of inner retinal layers and RPE irregularities. Large foveal ORT (OD), perifoveal ORT (OS). Hyper-reflective deposits above the RPE	n/a

13	Peripheral bone spicules, retinal vessel attenuation, optic disc pallor, macular clumped pigmentation	Peripheral and macular hypo/hyper-AF mottling. Peripapillary hypo-AF, hyper-AF between the peripapillary area and the fovea. Diffuse central macular hypo-AF, and a central hyper-AF dot	Epiretinal membrane. Widespread loss of outer retinal layers, thinning of inner retinal layers with some foveal preservation. RPE irregularities. Hyper-reflective deposits above the RPE	n/a

14	Peripheral isolated bone spicules, retinal vessel attenuation, generalized hypopigmented dotting, optic disc pallor, circumscribed macular atrophy	Perivascular hypo-AF mottling. Hyper-AF macular ring surrounding a circumscribed central macular hypo-AF area	Epiretinal membrane. Widespread loss of outer retinal layers. Hyper-reflective deposits above the RPE	n/a

ELM, external limiting membrane; EZ, ellipsoid zone; n/a, not available; OD, oculus dexter; ORT, outer retinal tubulations; OS, *oculus sinister*; OU, *oculus uterque*; RPE, retinal pigment epithelium.

**Table 3:** Full-field stimulus threshold test results (dB).

Subject#	Blue stimulus		Red stimulus		White stimulus	
	OD	OS	OD	OS	OD	OS
2	59	57	35	25	26	26
3	52	51	41	37	30	30
6	53	53	23	24	24	26
12	64	55	31	28	26	25
13	36	28	35	26	26	19

**Table 4:** *PCARE* variants identified in the cohort.

Variant	Allele count in our cohort	Type	Classification (ACMG)	Criteria (ACMG)	Reference
c.551A > G (p.His184Arg)	4	Missense	LP	PM2, PM3, PP3, PP5	Novel
c.947del (p.Asn316Metfs^*∗*^7)	2	Frameshift	P	PVS1, PM2, PP5	[9]
c.1837C > T (p.Arg613^*∗*^)	6	Nonsense	P	PVS1, PM2, PP5	[11]
c.2950C > T (p.Arg984^*∗*^)	8	Nonsense	P	PVS1, PM2, PP5	[14]
c.3048_3049del (p.Tyr1016^*∗*^)	1	Nonsense	P	PVS1, PM2, PM3, PP5	Novel
c.3314_3315del (p.Ser1105^*∗*^)	1	Nonsense	P	PVS1, PM2, PM3	Novel

LP, likely pathogenic; P, pathogenic. (NM_001029883.3).

## Data Availability

All the clinical and molecular data that support the findings of this study are available from the corresponding author upon reasonable request.

## Supplementary Materials

Supplementary Materials Supplementary Table: Full Field ERG Raw Data showing microvoltage values in analyzed individuals.
