# Supplementary material for: Mutational Profile and Retinal Phenotypes of PCARE-Related Cone-Rod Dystrophies in a Mexican Cohort
Source: J Ophthalmol. 2024 Mar 4;2024:4003914. doi: 10.1155/2024/4003914 (PMC10927338; doi:10.1155/2024/4003914)
Supplement: Supplementary Materials — Supplementary Table: Full Field ERG Raw Data showing microvoltage values in analyzed individuals. [file 4003914.f1.docx]

| Full Field ERG Raw Data | | | | | | | | | | | | |
| --- | --- | --- | --- | --- | --- | --- | --- | --- | --- | --- | --- | --- |
|  | | **.01** | | **0.1** | | **0.3** | | **10** | | **3.0** | | **Flicker** |
| Patient # | **Eye** | **A μV** | **B μV** | **A μV** | **B μV** | **A μV** | **B μV** | **A μV** | **B μV** | **A μV** | **B μV** | **A μV** |
| 2 | **OD** | -1.6 | 3.1 | -14.8 | 8.5 | -5.5 | 4.7 | 5.4 | -10.1 | 1.4 | 1.0 | -4.7 |
|  | **OS** | -3.9 | 6.1 | -9.4 | 5.5 | -3.9 | 11.6 | 3.0 | -8.6 | -1.7 | 4.2 | -3.4 |
| 3 | **OD** | -4.7 | 7.7 | -3.1 | 6.1 | -2.3 | 4.5 | -2.3 | 6.1 | -1.6 | 2.3 | -0.9 |
|  | **OS** | -3.1 | 4.6 | -4.7 | 7.7 | 1.5 | 9.3 | 3.8 | 7.0 | 1.5 | 9.3 | -0.1 |
| 6 | **OD** | 0 | 0 | 0 | 0 | 0 | 0 | 0 | 0 | 0 | 0 | 0 |
|  | **OS** | 0 | 0 | 0 | 0 | 0 | 0 | 0 | 0 | 0 | 0 | 0 |
| 7 | **OD** | -10.2 | 66.4 | -3.1 | 74.1 | -14.1 | 77.3 | -54.7 | 101.5 | -1.9 | 5.7 | -2 |
|  | **OS** | -13.3 | 82.7 | 0 | 77.2 | -46.9 | 60.9 | 64.1 | 95.3 | -6.1 | 6.7 | -9.1 |
| 8 | **OD** | -7.8 | 5.5 | 2.2 | 6.3 | -7.8 | 8.6 | -3.9 | 6.1 | -5.7 | 6.4 | 0 |
|  | **OS** | -6.3 | 7 | 0 | 0 | 0 | 0 | -1.6 | 22.6 | -2.1 | 4.2 | 0 |
| 9 | **OD** | -0.8 | 8.5 | -5.5 | 4.7 | 0 | 0 | -8.6 | 8.6 | -2.8 | 6 | -16.4 |
|  | **OS** | 0 | 0 | 0 | 0 | 0 | 0 | 0 | 0 | -1.2 | 4.7 | -9.5 |
| 10 | **OD** | -1.6 | 4.6 | -3.1 | 6.1 | 0 | 0 | 0 | 0 | -3.3 | 3.4 | -3.1 |
|  | **OS** | -1.6 | 4.6 | 0 | 0 | -3.1 | 5.3 | 0 | 0 | 0 | 0 | -6.3 |
| 11 | **OD** | 0 | 0 | 9.3 | 35.9 | -10.9 | 37.4 | 35.9 | 42.8 | -4.9 | 5.6 | -4.3 |
|  | **OS** | -3.9 | 37.4 | -5.5 | 34.3 | -7.0 | 35.8 | -21.9 | 40.6 | -6.3 | 4.7 | 0 |
| 12 | **OD** | -11.7 | 13.9 | -20.3 | 24.9 | 1.5 | 12.5 | 0 | 0 | 0 | 0 | 0 |
|  | **OS** | 1.5 | 1.5 | -11.7 | 4.7 | 0.7 | 6.2 | -10.2 | 11.7 | -1.0 | 1.6 | 3.5 |
| 13 | **OD** | -3.9 | 13.2 | 0 | 0 | -0.8 | 5.4 | -3.1 | 3.8 | -0.8 | 1.9 | -1.7 |
|  | **OS** | -25.8 | 21.1 | -11.7 | 14.7 | 0 | 0 | -6.3 | 11.7 | 0 | 0 | 0 |

*Raw microvoltage values were taken using approximate milisecond data for the identification of “a” and “b” waves respectively.

*Patient 6 showed artifact interference due to fixation absence.
